# The Specificities of Elite Female Athletes: A Multidisciplinary Approach

**DOI:** 10.3390/life11070622

**Published:** 2021-06-26

**Authors:** Carole Castanier, Valérie Bougault, Caroline Teulier, Christelle Jaffré, Sandrine Schiano-Lomoriello, Nancy Vibarel-Rebot, Aude Villemain, Nathalie Rieth, Christine Le-Scanff, Corinne Buisson, Katia Collomp

**Affiliations:** 1 CIAMS, Université Paris-Saclay, 91405 Orsay, France; carole.castanier@universite-paris-saclay.fr (C.C.); caroline.teulier@universite-paris-saclay.fr (C.T.); sandrine.schiano-lomoriello@univ-orleans.fr (S.S.-L.); nancy.rebot@univ-orleans.fr (N.V.-R.); aude.villemain@univ-orleans.fr (A.V.); nathalie.rieth@univ-orleans.fr (N.R.); christine.le-scanff@universite-paris-saclay.fr (C.L.-S.); 2 CIAMS, Université d’Orléans, 45067 Orléans, France; 3 LAMHESS, Université Côte d’Azur, 06205 Nice, France; valerie.bougault@univ-cotedazur.fr; 4 B3OA, Université de Paris, 75010 Paris, France; christelle.jaffre@u-picardie.fr; 5 Département des Analyses, AFLD, 92290 Chatenay-Malabry, France; c.buisson@afld.fr

**Keywords:** menstrual phase, amenorrhea, hormonal contraception, performance, health, Athlete Biological Passport

## Abstract

Female athletes have garnered considerable attention in the last few years as more and more women participate in sports events. However, despite the well-known repercussions of female sex hormones, few studies have investigated the specificities of elite female athletes. In this review, we present the current but still limited data on how normal menstrual phases, altered menstrual phases, and hormonal contraception affect both physical and cognitive performances in these elite athletes. To examine the implicated mechanisms, as well as the potential performances and health risks in this population, we then take a broader multidisciplinary approach and report on the causal/reciprocal relationships between hormonal status and mental and physical health in young (18–40 years) healthy females, both trained and untrained. We thus cover the research on both physiological and psychological variables, as well as on the Athlete Biological Passport used for anti-doping purposes. We consider the fairly frequent discrepancies and summarize the current knowledge in this new field of interest. Last, we conclude with some practical guidelines for eliciting improvements in physical and cognitive performance while minimizing the health risks for female athletes.

## 1. Introduction

The impact of intensive physical practice on women has attracted substantial attention in recent years. Yet, as women have been performing at the international level for just half a century in most sports disciplines, knowledge specifically on the elite female athlete has lagged behind that on elite male athletes. Indeed, one might say that in this sense, there is no real equality between women and men in sport [1]. A notable point in this regard is that the physiological (i.e., medical, dental, nutritional, and biological) and psychological monitoring of high-level athletes was established for male athletes and simply extrapolated to female athletes. More importantly, these extrapolations were made without serious questioning of the validity of this transfer [2], and the effects of the menstrual cycle phases, amenorrhea, the intake of oral estroprogestative or progestin-only pills, and intrauterine devices on female athletes are almost never taken into account. This is surprising, given their likely impacts not only on the physiological and psychological stress status of athletes but also on responses and adaptations to exercise [3].

The specificity of the female athlete is linked in part to the menstrual cycle (MC), with cycle length varying from one woman to another, although generally between 22 and 32 days (28 days on average). The first day of the cycle is the beginning of menstruation (day 1). The follicular phase (F) is the first phase of the normal menstrual cycle (NMC) before ovulation (from day 1 to around day 14, in the middle of the cycle), and the luteal phase (L) is the phase after ovulation until the first day of the next menstruation. The cycle is described by the fluctuation of the ovarian hormones: estrogens and progesterone. The estrogen leader, estradiol (E2), increases in both F and L, thus showing two peaks in concentration: one at the end of F (LaF), with a peak at the peri-ovulatory time (PeO), and one in the mid-luteal phase (MiL). Progesterone (PG) increases only once with a peak in L concomitant with the second E2 peak. The gonadotropins produced by the pituitary gland, follicle-stimulating hormone (FSH) and luteinizing hormone (LH), peak during the ovulation phase, with during the late luteal phase (LaL) and the menstrual period (early follicular phase, EaF), levels of E2 and PG at their lowest. The menstrual cycle often appears to be disrupted in high-level athletes. First, even with regular cycles, female athletes can have prolonged F with shortened or absent L, secondary to a deficit in LH that induces insufficient PG production [4]. Moreover, a significant number of these athletes show disorders ranging from oligomenorrhea to amenorrhea (AM). Oligomenorrhea is defined as long and/or irregular MCs or cycles longer than 32 days [5], whereas AM is the absence of menstrual periods. Their prevalence in the general population is about 2 to 5% but increases among athletes, especially AM, ranging from 3.4% to 70%, with the highest prevalence observed in sports where the fat mass is very low, such as dancing and long-distance running [6,7,8]. The origin of AM, which is directly linked to a high workload and/or insufficient food intake, is probably multifactorial, with other main factors being (i) excessive secretion of endorphins, inducing a high opioid tone inhibiting the hypothalamic-pituitary-gonadal axis with subsequent inhibition of LH, FSH, E2, and PG secretion; (ii) insufficient fat mass to prevent the transformation of androgens to estrogens with a parallel decrease in leptin; and (iii) excessive secretion of prolactin (PRL).

Between 20 and 70% of elite female athletes use hormonal contraception, with a significant variation depending on the country (Australia, Denmark, Norway, Sweden, United States) and the sports discipline (football, basketball, track and field…) [7,9,10,11]. A large majority (68–75%) of these athletes take second-generation combined estrogen-progestin oral contraceptives (COCs), rather than progestin-only contraceptives (20–30%) [7,10,11]. The mono-, bi-, and triphasic COCs contain ethinylestradiol as the estrogen at concentrations between 20 and 50 μg per pill and, most of the time, levonorgestrel or norgestrel as the progestin at concentrations of 50 to 150 μg and 500 μg, respectively. They are taken for 21 days followed by 7 days off to reproduce the MC, but without the E2 and PG peaks and thus with more stabilized hormone levels. COCs are known to significantly decrease both free and total blood testosterone (TES) [12]. Progestin-only contraception is used continuously, either in tablet form or with an intrauterine device (IUD), and the main progestins are levonorgestrel and desogestrel. A significant number of elite athletes use hormonal contraception primarily to regulate their MC (24%), particularly those with a history of menstrual disturbances [9]. Moreover, a majority of COC users report having deliberately manipulated timing, frequency, and amount of menstrual bleeding, with a prevalence of 60–77% among competitive athletes, who most often cited special events and holidays, convenience, and sports competition as the reasons [7,11,13]. Non-COC users and, to a lesser degree, COC users report negative side effects during their MC, primarily in LaL with premenstrual syndrome (PMS) and during menstruation with dysmenorrhea, defined as pain during days 1–2 of menstruation.

Recent reviews [14,15] have examined the impact of MC and COC administration on physical performances in women ranging from sedentary to elite and from young to premenopausal, but the intention of this review is to focus on the specificities of the elite female athlete from a multidisciplinary perspective. We first present the current but still limited data on the effects of MC, AM, COC, and other hormonal contraceptives on both the physical and cognitive performances of these elite athletes. To highlight the mechanisms, as well as the potential performances and health risks in this population, we then take a multidisciplinary approach and present the causal/reciprocal relationships between hormonal status and mental and physical health in young (18–40 years) healthy females, both trained and untrained. We thus cover the research on food intake, body composition, bone and ligament; the central nervous system (CNS) and neuromuscular and muscular responses, from decision-making to motor achievement; cardiorespiratory, gastrointestinal, urinary, endocrine, metabolic, and immune responses; and the Athlete Biological Passport (ABP) used for anti-doping purposes. We consider the fairly frequent discrepancies in the study results and summarize the current knowledge in this new field of interest, underlining the potential effects of hormonal status on both biological and psychological functioning and assessing the implications of these effects. Last, we conclude with some practical guidelines and perspectives for eliciting maximal improvements in physical and cognitive performances in female athletes while minimizing the health risks.

## 2. Hormonal Status and Performance in Elite Female Athletes

### 2.1. Hormonal Status and Physical Performance

A recent systematic study [14] explored the responses of sedentary to highly trained NMC women, without addressing the effect of age, and showed that exercise performance tended toward but was not significantly reduced during EaF compared to other MC phases. However, we cannot extrapolate the performance results obtained in this large cohort that included sedentary, active healthy or leisure sports women, and elite athletes, and thus we will only target this specific population.

#### 2.1.1. Elite NMC Athletes

- Short intense exercise

Knee flexion and extension peak torque, passive knee joint position sense, and postural control [16] assessed in collegiate female athletes were similar between mid-follicular (MiF), PeO, and MiL. Sprinting performance in trained female students involved in multiple sprint and power events [17] were not different in MiF, PeO, and MiL, with the same metabolic responses, i.e., blood lactate, blood pH, and plasma ammonia. In parallel, no MC effect on jumping or sprint performance was found in high-level female soccer players [18,19] in EaF, LaF, and L. The same muscle strength, force, velocity, and anaerobic performance during a Wingate anaerobic test in female kickboxing athletes and judokas were also reported across MC [20,21,22]. However, the number of throws in the first 15-s period of a specific judo test in judokas was better in L [21], whereas a study [23] showed significant decreases in peak torque production of the extensors and flexors across isokinetic exercises in EaF vs. other MC phases in well-trained females.

- Incremental maximal test and endurance exercise

No modification in performance between MC phases at VO_2_max and peak treadmill velocity was found in endurance athletes, with tests performed either in EaF, LaF, EaL, and LaL [24] or in MiF and MiL [25]. On the field, the same results were obtained in elite soccer players by Tounsi et al. [19] but not by Julian et al. [18], who reported a reduction in performance in MiL during the Yo-Yo test. Last, Kishali et al. [26], using a questionnaire, estimated that physical performance was not affected during MC for most female athletes even in EaF, with decreased menstruation pain during training and competition.

#### 2.1.2. Elite AM Athletes

The direct impact of AM on performance in elite athletes has almost never been investigated with, to our knowledge, only two direct studies. Vanheest et al. [27] examined the impact of ovarian suppression on the performances of junior elite female swimmers and showed that AM athletes with an energy deficit had a regression in their 400-meter swim velocity over 12 weeks compared to NMC athletes who showed an improvement. In parallel, they reported that E2, PG, triiodothyronine (T3), insulin-like growth factor (IGF-1), and energy status markers were highly correlated with sports performance. Similarly, Tornberg et al. [28] found lower neuromuscular performance in AM vs. NMC elite endurance athletes, with AM athletes showing longer reaction times and decreased knee muscle strength and endurance. This lower neuromuscular performance was positively correlated with lower fat-free mass, glucose, E2, and T3, and negatively correlated with cortisol levels. In an indirect manner, Ackerman et al. [29] assessed the responses to an online questionnaire completed by 1000 female athletes from 15 to 30 years old and reported that athletes with low energy availability were more likely to be classified as having an increased risk of menstrual dysfunction combined with low performance. Indeed, athletes with low vs. adequate energy availability were 2.1 and 1.5 times more likely to report increased recovery time and decreased endurance, respectively, whereas decreased muscle strength, which is difficult to quantify via self-report, was not queried.

#### 2.1.3. Elite COC Athletes

- Short intense exercise

Studies with monophasic [23,30] or triphasic [31] COCs have reported no change in anaerobic speed or isokinetic strength in highly active women or university athletes, whatever the progestin used. In parallel, Rechichi et al. [32] showed similar anaerobic power and repeat sprint ability in team-sport athletes at three time points of a single COC cycle, i.e., consumption, early and late in the withdrawal phase [32]. In the same way, swimming performance during a 200-m time trial was not affected within a monophasic COC cycle in competitive swimmers and water polo players at the same three time points of the COC cycle [33], in addition to unchanged body composition, peak heart rate, and blood glucose. However, peak blood lactate was significantly lower and mean pH higher late in the withdrawal phase, coupled with higher endogenous E2 levels and an increase in pH, possibly because of an increase in fluid retention, plasma volume, and cellular alkalosis. Yet, other data were discrepant. Redman et al. [34] reported in female rowers during the COC cycle an improved peak power output and anaerobic capacity with low estrogen and progestin, coupled with higher plasma glucose concentrations and lower plasma triglyceride concentrations at rest and during exercise. In the abovementioned study, Rechichi et al. [32] nevertheless reported decreased performance late in the COC withdrawal phase for reactive strength, which was interpreted as a negative impact of endogenous estrogen on neuromuscular timing and muscle activation time.

- Incremental maximal test and endurance exercise

No change in aerobic performance was found in trained runners with a monophasic [35] or a triphasic [31] COC despite a 4.7% decrease in absolute and relative VO_2_max and an increase in fat mass with COC vs. EaF [31]. Rickenlund et al. [36] reported that endurance athletes after 10 months of monophasic COC use a significant increase in weight and fat mass only in athletes with previous oligo-/AM, associated with a decrease in ovarian androgens and increased bone mineral density (BMD), with the largest increase in the athletes with low BMD at baseline. Despite the significant changes in body composition, no significant impact on physical performance was recorded. Last, Rechichi et al. [37] found with a monophasic COC no difference in endurance performance in female cyclists/triathletes despite greater ventilation, oxygen equivalent, and blood lactate values during hormonal vs. withdrawal phase. In contrast, Sunderland et al. [38] examined the impact of COC use on the performance of high-intensity intermittent running in the heat in well-trained athletes twice within the COC cycle. Heart rate (HR), perceived exertion, sweat rate, plasma lactate, and ammonia did not differ, but the rectal temperature was higher, and performance was improved at days 15–28 vs. days 1–14.

### 2.2. Hormonal Status and Cognitive Performance

#### 2.2.1. Elite NMC Athletes

Burrows et al. [24] found no modification in negative and positive affect and irritability in female runners between EaF, LaF, EaL, and LaL. In contrast, Cockerill et al. [39] used the profile of mood state (POMS) questionnaire in elite NMC runners vs. inactive women 5 days before and 14 days after menstruation and reported that all athletes were less tense, depressed, tired, confused, and angry and more vigorous in PeO than in LaL, but with lower difference than non-trained subjects. It is interesting to note that the lower-intensity-training runners appeared to benefit psychologically, while high-intensity training had an adverse effect on mood. Moreover, PMS appeared to cause marked negative mood swings. Other studies indirectly investigated changes in self-reported physical fitness, performance, and side effects across MC, using various questionnaires given to the athletes, and all reported consistent results [11,26,40,41,42,43]. Indeed, 50% to 80% of the elite athletes claimed they were affected by PMS in the week prior to menstruation, and about 80% of them by menstruation. Most perceived their worst performance as being close to bleeding and scored highest for perceived fitness and performance in the phase following bleeding [43]. Forster et al. [44,45] showed that female soccer players with PMS had increased anxiety and pro-inflammatory cytokines compared to athletes without PMS and that this was combined pre-game with, respectively, greater tension in F and greater depression in L. Findlay et al. [46] conducted semi-structured interviews with elite female rugby players and focused on MC-related issues such as symptoms, the perceived impact of menstruation on different aspects of daily living and performance, methods of dealing with menstruation-related concerns, and available support and comfortability. Almost all athletes (93%) reported MC-related symptoms, with high inter-individual responses. As in the studies of Bruinvels et al. [41,42], one-third perceived heavy menstrual bleeding, and two-thirds reported symptoms that impaired their performances and self-medicating to alleviate the symptoms. Bruinvels et al. [42] therefore proposed a novel menstrual symptom index (MSi) to quantify their frequency.

#### 2.2.2. Elite AM Athletes

The abovementioned study of Cockerill et al. [39] included AM athletes, and the data of the POMS used with a 21-day interval showed little variation between the two periods. In a comparison of elite AM and NMC athletes, Ackerman et al. [29] showed that athletes with increased risk of menstrual dysfunction were more likely to have impaired judgment, decreased coordination, decreased concentration, irritability, and depression, coupled with altered memory performance [47], this last effect probably mediated by the lack of E2, as estrogen replacement improves verbal memory and executive control in this population [48].

#### 2.2.3. Elite COC Athletes

Brown et al. [40] found that the decision to actively control the MC was often triggered by a desire to reduce the effect on competition, to lessen anxieties about meeting the required weight, or to reduce the distractions to manage during competition. Oxfeld et al. [7] studied self-perceived physical and emotional symptoms related to COCs in elite female athletes and reported that their use did not abolish dysmenorrhea but may have reduced emotion-related side effects. For their part, Martin et al. [11] reported that the most common side effects after hormonal contraception were weight gain and mood swings, both of which can affect performance. It can also be noted that negative side effects are more common with progestin-only vs. COC users, especially with the IUD.

## 3. Possible Mechanisms: Performance and Health Risks

### 3.1. Food Intake, Body Composition, Bone, and Ligament

#### 3.1.1. Food Intake and Body Composition

- NMC women

Some studies have shown significant variations of appetite and energy intake in healthy women across their MC [49,50], in part explained by the influence of E2 and PG on gastric emptying and the secretion of gastrointestinal hormones such as glucagon-like-peptide-1 (GLP-1) and cholecystokinin (CCK) [49]. Food intake thus may be higher during L than F in lean women [51,52], with an increase in macronutrient intake, especially sweet foods, in LaL [53,54], but the changes remain poorly understood. Female athletes have distinct nutritional needs based on their sport, exercise intensity, and season [55], with disordered eating observed in athletes from several sports, especially esthetic ones [56], limiting understanding of the impact of MC on food intake. No difference in body mass or composition, determined by dual-energy absorptiometry (DXA) [57] or bioelectrical impedance [58], was found in well-trained NMC women across MC in most studies, with one study shows, however, an increase in body mass in L vs. F in the more hydrated and slim women [59].

- AM women

Classically, disordered eating, menstrual dysfunction, and low bone mass have been grouped under the term female athlete triad (FAT). This concept was recently renamed: relative energy deficiency in sport (RED-S), as proposed by the International Olympic Committee (IOC). The use of this much broader term is linked to the observation that the main problem seems to be the lack of available energy, with consequences affecting several systems, as a “spectrum” of occurring irregularities [60]. All papers have reported significantly lower fat mass in AM vs. NMC athletes, coupled with decreased fat-free mass in most of them [28,61,62,63,64,65].

- COC women

Nutritional choices do not seem to be adjusted to the use of COC [66], but the current data are insufficient to conclude on whether COC is associated with changes in dietary behavior. In addition, COC use was reported not to alter [58,67] or to increase body and fat mass [30,68], generally limited to athletes with previous oligo-/AM [36].

#### 3.1.2. Bone Health and Ligament Injury Risk

The constant bone remodeling occurs under the control of mechanical loading, cytokines, growth factors, thyroid, and sex hormones. Estrogens are considered as the “key regulator” of bone metabolism in women [69], positively affecting the formation and proliferation of bone-forming osteoblasts and simultaneously inhibiting apoptosis of bone-resorbing osteoclasts, although the influence of PG should not be completely overlooked. Indeed, a recent study [70] indicated that E2 exerts an immediate positive effect on the production of osteoprotegerin, a bone formation marker, while PG seems to have a delayed positive effect.

- NMC women

Bone mineral accrual in adolescence and young adulthood is determined by MC, with optimal estrogenic impregnation essential to provide the high peak bone mass that can lower fracture risk later in life [71,72,73,74,75]. Bone formation assessed by osteocalcin and bone resorption assessed by type I carboxyterminal telopeptide (ICTP) did not change significantly across MC in NMC female rowers [57]. With regard to training, a systematic review on the influence of sports practice on BMD and bone geometry in healthy children and adolescents [76] highlighted that female gymnastic, soccer, tennis, and capoeira practitioners have better values than control subjects and comparisons of female soccer players and swimmers showed that sports requiring impact and body overloading promoted BMD content [76,77]. In addition, women injure the knee anterior cruciate ligament (ACL) four to six times more often than men, which has prompted the suggestion that fluctuations in women’s endocrine environment might be a factor [78]. The presence of sex hormones or relaxing receptors in ACL might explain their effect on this structure, but other extrinsic or intrinsic factors may contribute to this process [79,80]. Recent reviews [81,82] seem to show a decreased risk of ACL injury during L vs. F and PeO, with increased ACL laxity in PeO at high E2 levels vs. other MC phases. However, no association was found between increased risks of ACL tears and increased laxity [82]. Moreover, other authors have reported no significantly different ACL laxity in high-level female athletes in F, PeO, and L, at rest or during exercise [83,84].

- AM women

Menstrual dysfunction in female athletes and delayed onset of menarche in adolescent girls have been associated with lower BMD [85,86], which can, however, be modulated by physical training. Indeed, heavy weight-bearing training in sports is associated with higher bone mass in the athletes compared to controls with the same age and menstrual status and could moderate the decrease in bone mass in AM athletes [87], although residual deficits seem to persist without catch-up raising concerns for suboptimal peak bone mass acquisition [88]. Moreover, the favorable action of training appears attenuated in sports practices that exert lower strain [89]. Last, the data are sparse, but menstrual dysfunction may increase the risk of ACL injury [78].

- COC women

The effects of COCs vary more widely, and factors such as concentration in estrogens and/or progestins, age of the studied population, age of initiation of COC use, and physical aptitude may explain the differences between studies [89,90]. A meta-analysis [91] of studies on non-athletic women over 30 years old using a range of COCs reported either positive or no effect on women’s bone health, with most studies reporting no change in spine, hip, or total body BMD between COC users with various doses and progestins and non-users [92,93]. However, other authors [94,95] have noted that young females who initiate COC use early after menarche may experience skeletal detriments. Last, Martin et al. [70] observed no significant difference in the bone formation marker level between COC phases, whereas the resorption marker level was significantly lower in the last vs. the early phase of COC consumption. In female athletes, Jürimäe et al. [57] reported decreases in both osteocalcin and ICTP in COC vs. NMC. Most of the other studies have been conducted in oligo-/AM athletes, and the data indicated either a slight gain in BMD, essentially in the lumbar spine after six months of COC use [96], or tendencies without real gain [97]. Recently, the impact of the route of estrogen administration on bone turnover markers in oligoamenorrheic athletes and its mediators [98] was demonstrated, and Ackerman et al. [99] reported lumbar spine and femoral neck BMD improvement with transdermal estradiol plus cyclic oral progesterone but not with COC administration in oligo-/AM athletes. In parallel, different studies have suggested a potential 20% reduction in risk of ACL rupture under COCs [81,100,101]. It was hypothesized that the relative progestative dominance in COCs mitigates the estrogen effect [81]. However, all the authors noted small sample sizes, the heterogeneity of the subjects, and methodological concerns as study limitations [102]. Last, Nose-Ogura et al. [103] showed that COCs decreased serum relaxin-2 levels in athletes with high relaxin-2 concentrations during L. This may be of interest as recent investigations have demonstrated that these athletes have a high risk of ACL injuries, but the mechanism remains to be explained.

### 3.2. CNS and Neuromuscular Responses (Part 1): Decision Making

A substantial body of literature in neuroscience shows that sex steroid hormones influence cognitive processes [104,105,106]. The clearest evidence of the direct neurological effects of E2 and PG was the discovery of their receptors in multiple brain sites. Both E2 (ERα and ERβ) and PG receptors (PR-A and PR-B) have been found in brain areas associated with reproduction, but also in brain areas involved in cognitive functions and emotional processing, such as the hypothalamus, amygdala, hippocampus, and prefrontal cortex [107,108,109,110]. E2 and PG also act as modulators for other neurotransmitter systems, including the cholinergic, serotonergic, GABAergic, dopaminergic, and glutamatergic pathways, all of which play roles in executive function, learning, memory, or reward processing [111,112,113].

#### 3.2.1. Cognitive Function

- NMC women

A decades-old theory postulates that men perform better in visuospatial skills while women outperform in verbal skills [114]. This theory was applied to MC with the assumption that these “sexually dimorphic” cognitive abilities/skills would differ across MC [115]. It was therefore expected that women would perform better on visuospatial tasks during phases with low E2 and PG levels and that, in contrast, performance on verbal tasks would be improved during LaF and MiL; in view of recent critical reviews, evidence is nevertheless insufficient to support the sexually dimorphic hypothesis [116,117,118,119,120]. Indeed, regarding visuospatial ability, only a few studies on mental rotation, the most commonly used test [120], have supported the hypothesis of superior performance in EaF [121,122,123], whereas the others showed no significant findings [124,125,126]. Moreover, on the other visuospatial abilities (including spatial perception, spatial navigation, and spatial math tasks), only some reported improved performance in F vs. L [122,127]. Other studies established no MC influence on visuospatial memory or ability or reported findings conflicting with the hypothesis [125,128,129,130]. The two main cognitive domains identified as favoring women [131] are verbal fluency and verbal memory [120], but other verbal skills such as semantic retrieval, implicit verbal memory, and verbal working memory have also been studied. Although some studies showed improved verbal task performances in LaF or MiL [123,128,132], most found no effect of the MC phase on verbal skills or reported findings conflicting with the sexually dimorphic hypothesis [122,124,125,133,134,135,136]. MC studies have also examined executive functioning tasks that require diverse higher-order abilities such as judgment, planning, and control of other domains. Again, the studies were inconclusive. Indeed, one study on flexible thinking and problem-solving showed better performance in EaL [136], whereas another study observed no MC effect [130]. Results on inhibition and selective attention performance varied, being either impaired or improved in LaF and improved or unimproved with high E2 levels, depending on the task [133,137,138]. Two studies on spatial memory suggested that high E2 levels were related to improved performance [139,140].

- COC women

A recent systematic review showed that the impact of COCs on cognitive abilities remains controversial [141]. Indeed, the studies investigating associations between COC use and visuospatial abilities have shown mixed results, with most reporting no significant difference between COC users and NMC women [124,125,142,143]. However, other studies, using a variety of tasks and a broad range of COC formulations, have shown improved visuospatial ability [144,145,146], whereas others have shown impairment [124,147]. Wharton et al. [148] analyzed the cognitive impact of COC subtypes and found no overall difference in visuospatial ability between COC users and non-users. However, they showed that the new-generation pills with anti-androgenic progestins impaired visuospatial ability, whereas androgenic progestin COCs improved it compared to NMC women [149] and suggested that the opposing effects of anti- and pro-androgenic progestins may potentially mask each another when COC subtypes are examined collectively, which may explain the inconsistencies in the literature. Data on the impact of COC on verbal memory are also discrepant, with either no change [143,148,150] or improvement [125,141,142] both in comparison to NMC women and across the COC cycle. When improvement occurs, this is likely attributable to the effect of ethinylestradiol [104,123]. Studies have also investigated the COC effects on other verbal skills, with inconsistent findings. Only one study found that COC use decreases verbal fluency [124], with this negative impact more pronounced in androgenic COC formulations, whereas other studies showed no significant effects of COC use [125,143,144]. Most studies on executive function, attention, and working memory have found no significant effect of COC use [125,143,151], but some have reported improvements in attention, concentration, and working memory [142,152,153]. As with most research in this area, there has been no sub-analysis with respect to the impact of the progestin.

#### 3.2.2. Emotions and Social Interactions

Few studies have explored the impact of hormonal status on emotions and social interactions in elite athletes, but a number of studies have done so with a larger young, healthy female population.

- NMC women

Whereas PeO with its high E2 level is generally associated with a positive mood and high levels of well-being and self-esteem [154], pre-menstruation and menstruation are often associated with a negative mood, with direct links to low E2 and PG. During F, women have a low level of negative affect, which increases during L and reaches a paroxysm at the time of pre-menstruation. With regard to social relations, Derntl et al. [155] put forward the idea that women discriminate emotions more easily in F than in L, suggesting that the levels of empathy and social sensitivity differ with the phase of MC. Reynolds et al. [156] showed that women with higher average PG levels reported higher anxiety levels than women with lower PG, coupled with a greater increase in anxiety across MC, providing support for a link between PG levels and subjective anxiety. Numerous studies have reported that from 30% to 95% of women experience undesirable premenstrual mood changes during LaL, characterized by symptoms such as tension, irritability, restlessness, anxiety, and depression, with a positive correlation between pain and states of anxiety and depression [157,158,159]. Menstruation is relatively often accompanied by dysmenorrhea in healthy young women, which may have emotional consequences, including sleep disturbance, daytime fatigue, and drowsiness [160,161,162,163,164]. Subjective sleep quality appears to be lowest around menses, especially in women suffering from PMS [165,166,167], but the clinical significance was found to be no longer statistically significant when potentially confounding variables such as perceived stress, and social support were included [168,169]. In addition, MC had an impact on psychosocial measures in all women, with and without PMS, in F and L, but PMS was associated with more reports of daily stress and social conflict. These negative emotional effects have been shown to decrease quality of life, impacting personal and professional relationships, as well as school or work performance and absenteeism [170,171,172,173,174,175].

- COC women

Recent articles [176,177,178] have reviewed the current knowledge on how COCs affect mood. COCs seem to reduce the variations across MC, whether or not the premenstrual mood is specifically improved [176,177,179,180]. Ekenros et al. [179] reported that the onset of COC use significantly decreased PMS but did not affect mood symptoms, whereas Robakis et al. [177] showed that some women experienced the beneficial effects of COCs specifically on premenstrual mood symptoms. A recent review [178] reported that COC use in most women shows no effect or a beneficial effect on mood, with the fewest mood effects obtained with the less androgenic progestins and continuous or non-oral COCs. The links between mood and changes in androgens in women starting on COCs were also studied [180,181], with no relationship found, even though some women may be more sensitive to changes in TES than others. Last, women taking hormonal contraceptives had less slow-wave sleep than NMC women, with the longest mean nightly sleep time occurring with progestin-only oral contraception but with only minor consequences for the quality of sleep [182,183].

### 3.3. CNS and Neuromuscular Responses (Part 2): Motor Achievement 

The discovery of the impact of E2 and PG at every level of the CNS [184,185] down to the muscle itself [186] prompted researchers to investigate the potential effects of sex hormone variations on balance and motor control in women. Although the importance of estrogen action is unclear, postural control [187,188] and motor control [189,190] have been found to vary across the MC phases (Table 1).

#### 3.3.1. Postural Control

- NMC women

Many studies have shown variations in postural instability over the MC, but the results have been discrepant based on task difficulty mainly [16,187,188,191,192,193,194,195,196,197,198,199]. Overall, the researchers assumed that, as the main structures contributing to balance are influenced by hormonal fluctuations, differences in balance would be seen over MC unless adaptive mechanisms take place [200]. Two hypotheses have emerged based on when postural instability occurs in the course of MC. Researchers reporting less stability during PeO have argued that E2 triggers ligament laxity and weakens muscle strength [201,202,203] as it has an inhibitory effect on collagen synthesis [204,205]. Conversely, researchers who found more instability around menses have argued that hormonal changes taking place at the beginning and end of MC might affect many aspects of postural stability simultaneously rather than just one source of postural information, such as the vestibular system [187].

- COC women

Only a few studies have looked at COC women, showing stabilization of posture or a better balance for COC users [194,206,207]. The consistent lower limb dynamics of COC users might demand less reliance on the neuromuscular control apparatus to acutely alter feed-forward strategies during dynamic tasks. This may explain the lower rate of lower limb musculoskeletal injuries in this population compared to non-COC users [208], with a protective effect on knee injuries [206].

#### 3.3.2. Motor Control

- NMC women

Authors have found similarly mixed results, independently of the tested task or athlete level [18,19,209,210,211,212,213,214,215,216]. No variation in jumping or sprinting abilities across MC was reported [18,19], but in tennis, it should be noted that, although the speed of the serve did not vary, its accuracy decreased in PeO [212]. Many parts of the body implicated in motor control are known to be impacted by sex hormone variations, but whether the sum of those mechanisms is detrimental to the production of motor tasks remains unknown. In addition to the CNS changes described earlier, other factors might play a role in motor control regulation over the MC. Bayer et al. [211] suggested that functional cerebral asymmetries, probably due to hormonal modulations in interhemispheric interaction, could affect fine motor coordination across MC, with fluctuations in hand asymmetry. Ikarashi et al. [215] showed a failure of excitability of the primary motor cortex during L vs. PeO, resulting in weaker motor learning and motor performance. In terms of neuromuscular control, some researchers found various changes over MC, similar to findings in muscle co-activation patterns [192,233], whereas others reported no modification [209].

- COC women

The few studies on motor control have mainly found no variation in performance across the cycle in women taking COCs, but they also found no difference for non-COC users [217,218], making it difficult to conclude about a possible smoothing effect of COCs on motor abilities over MC based on the current literature.

#### 3.3.3. Strength and Resistance Training

- NMC women

MC effects on strength remain contradictory [30,219,220,221,222,223].

However, it seems that sex hormones can influence resistance training responses [186,224,225,226,234]. The gain in muscle strength might be due to a better process of satellite-cell incorporation-induced muscle hypertrophy, but further research is warranted, given the methodological issues and the lack of consensus, particularly on the potential alteration in skeletal muscle protein metabolism across MC [234].

- COC women

Older research found that COC use altered muscular strength [227,228] and an inhibiting effect on protein synthesis [229]. More recent papers, however, showed no difference in maximal force-generating capacity, jumping, or hopping with COC use [18,19,31,230] and did not suggest that COC use affects strength or endurance in response to strength-only or combined strength/endurance training [224,231,232]. However, further research is necessary to determine whether COC androgenicity plays a significant role in strength development [224].

### 3.4. Cardiovascular, Respiratory, Gastrointestinal, and Urinary Function

#### 3.4.1. Cardiovascular Response 

Some studies have examined the impact of female hormonal status on cardiac function and hemodynamics (HR, stroke volume, cardiac output, diastolic and systolic blood pressure) and autonomic control (heart rate variability: HRV, baroreflex sensitivity) (Table 2).

- NMC women

Studies have generally shown that resting cardiac function or hemodynamics does not depend on MC [235,236,237], but opposite results have been reported [168,238,239]. Lack of change in cardiovagal baroreflex sensitivity across MC was found by most [240,241,242] but not all [243] authors. In parallel, most but not all [244] studies have shown variations in HRV across MC, with an increase in the low-frequency peak (LF) in L, inducing a higher LF/HF ratio, coupled or not to a change in the QT interval or parasympathetic tone [245,246,247,248,249,250,251,252]. The alteration in the balance of E2 and PG may be responsible for the increased sympathetic activity in L, which appears, however, partly dependent on major lifestyle factors [253,254]. Last, circulating levels of cardiac natriuretic hormones did not show any significant change across MC in healthy women [255]. During exercise, most studies reported no change in stroke volume or cardiac output [237,256,257] in well-trained NMC athletes across MC, regardless of the environment, with two discrepant studies [258,259].

- AM women

No change was observed in the single study investigating the effect of menstrual disturbance on cardiovagal baroreflex sensitivity, vagal tone, and cardiovascular responses to orthostatic stress in young AM vs. NMC women in EaF [260], and the authors concluded that AM women do not display signs of impaired autonomic function or orthostatic responses.

- COC women

Early studies have shown higher systolic blood pressure and/or HR with COC use [235,261,262,263,264], but more recent studies did not report any change in either cardiac function or hemodynamics [236,262,265]. Most of the studies nevertheless did not note a difference in either HRV or baroreflex sensitivity after several months of COC use [236,262,266], with only one study showing impairment in autonomic control [267]. Last, the data suggest that progestin-only contraceptives do not modulate the autonomic balance [268]. Few studies have been conducted during exercise, and the data are discrepant, with either a greater increase in blood volume, stroke volume, and cardiac output during exercise with COCs [269] or no COC effects on HR [258] and cardiac vagal withdrawal at the onset of dynamic exercise [257].

#### 3.4.2. Respiratory Response

We first summarize here the current knowledge on the impact of hormonal status on respiratory parameters in healthy women in normal and hypoxic conditions. In addition, as most endurance athletes develop exercise-induced bronchoconstriction (EIB), often referred to as athletes’ asthma, we also report the data on the link between hormonal status and respiratory disorders, even though a recent systematic review suggested no link between sex and EIB [270].

- NMC women

E2 alone is not considered to have significant effects on the respiratory system [271,272]. In contrast, PG has been suggested to stimulate hyperventilation at rest and during exercise and to enhance respiratory drive at rest in women during L by acting centrally on hypothalamic sites [273,274,275] and through peripheral chemosensitivity [274]. Some studies have thus observed an increase in ventilation (VE) response at rest and during exercise in MiL in NMC non-athletes [276,277,278,279,280], although others have not [67,281,282,283]. No changes in lactate or ventilatory thresholds were reported [279]. Consistent with the increased VE, decreases in PaCO_2_ and PETCO_2_ and increases in carbon dioxide ventilatory equivalent (VE/VCO_2_) were observed during incremental exercise tests in NMC non-athletes in MiL [272], with PaCO_2_ negatively correlated with E2 and PG [278]. Dombovy et al. [284] reported no change in exercise VE in MiL but observed a significant decrease in VCO_2_ and thus an increase in VE/CO_2_. The change in VE is not expected to limit VO_2_max, and most works have shown no change in VO_2_max across MC at sea level or at 3600 m for high-altitude native women [219,285]. Nevertheless, an increase in the oxygen ventilatory equivalent (VE/VO_2_) ratio during submaximal exercise is sometimes observed in L [279].

In trained NMC females, an increase in tidal volume during L vs. EaF and MiF was reported [278], whereas no change in VE during maximal or submaximal exercise [257,258,276] or in VO_2_max was generally found across MC [258]. It has not been excluded, however, that VO_2_ depends on E2 concentrations or the E2/PG ratio, as Barba-Moreno et al. [258] recently showed higher VO_2_ in MiF than EaF during submaximal exercise in trained NMC females. A higher ventilatory drive in L than F has been found in most studies, as in non-athletes, but to a lesser extent, and without a significant performance impairment, likely due to the great variability in performance responses among female athletes [258,277,279]. Endurance athletes are known to have lower hypoxic and hypercapnic voluntary drive [286,287] and, not always coupled with the VE data, an increase in hypoxic and/or hypercapnic chemosensitivity has generally been observed at rest during MiL [277,284,288,289]. This contributes to reduced exercise tolerance, possibly through an increase in dyspnea perception [286,287,288,289,290]. However, other studies have found no difference in the ventilatory response to hypoxia at rest [281,283,291] or during exercise [284], probably due to the huge inter-variation in PG concentrations. Richalet et al. [292] recently observed that the increase in hypoxic ventilatory response during exercise was accompanied by higher exercise SaO_2_ during EaL and MiL and concluded that MC has an impact on the ventilatory response during exercise in hypoxia and, consequently, on tolerance to high altitude. This should be taken into consideration for women exercising in altitude. Last, few studies have investigated the potential MC impact on the susceptibility to upper respiratory tract infections (URTI) in NMC athletes, which remains under debate [293,294,295] despite the observation of the effect of an increase in estrogens on nasal mucosal immunity and nasal congestion in non-athletes [296].

Asthma and menstruation are closely linked, with menstrual symptoms correlated with the level of asthma control and its intensity in young women and irregular MC more frequently observed in asthmatics [297]. In NMC athletes, respiratory symptoms due to EIB or asthma, known as premenstrual asthma (PMA), worsen during L [298,299], increasing bronchodilator use during MiL vs. MiF. There was a negative correlation between the percent change in pre- to post-exercise FEV_1_ and PG but not E2 concentration. In line with these results, Oguzulgen et al. [298] found a significant increase in exhaled NO levels, induced sputum eosinophil counts, and daytime symptom scores, indicating a parallel worsening of airway inflammation during L, but Kirsch et al. [300] showed that E2 activates endothelial NO synthase in human airway epithelial cells via the epithelial estrogen receptor. On the other hand, many authors found that self-reported PMA in non-athlete women was associated with a decrease in peak expiratory flow during LaL, but they found no difference in forced vital capacity, FEV_1_ or PC_20_, with or without the increased use of inhaled β_2_-agonists [301,302,303,304].

- AM women

Ventilatory drive and exercise ventilation seem to remain stable in AM athletes [277] and are comparable to the F and L values observed in NMC athletes [256]. Shimizu et al. [295] observed more frequent URTI in AM compared to NMC distance runners and suggested that the low estrogen levels in AM might be related to higher susceptibility to infection in these athletes through a decrease in salivary IgA, which protects against the entry of pathogens into the body.

- COC women

In some but not all studies [67], monophasic COC use was found to increase VE, VE/VO_2,_ and breathing frequency in endurance athletes [37,258] during the hormonal vs. the early and late withdrawal phase, but this was not accompanied by a decrease in performance. In addition, Richalet et al. [292] showed that COCs had no effect on ventilatory responses to altitude hypoxia, whereas Regensteiner et al. [272] showed an increase in the hypoxic ventilatory response during mild exercise in L vs. F when progestin was used in normoxia. COC use may contribute to an aggravation of asthma by stimulating estrogen receptors in the bronchial epithelium [300,304]. Studies of the pro-inflammatory effects of COCs have indeed shown a strong increase in the plasma levels of C-reactive protein (CRP) and fibrinogen, but not interleukin-6 (IL-6) [305].

#### 3.4.3. Gastrointestinal and Urinary Function

Competitive sports activities are a clear risk factor for both gastrointestinal and urinary disorders in female athletes.

- Gastrointestinal function

Regular moderate physical activity is thought to protect against intestinal inflammatory diseases [306]. However, gastrointestinal symptoms (GIs) in the upper or lower gastrointestinal tract are common and a limiting factor for elite athletes, particularly during prolonged strenuous exercise, which can induce intestinal injury, increase permeability and endotoxemia, and impair gastric emptying, slowing small intestinal transit and causing malabsorption [307,308]. From 30% to 50% of athletes report GIs during training [309], with a prevalence reaching 70% in endurance disciplines [55,310,311]. Celiac disease and irritable bowel syndrome appear more prevalent among female than male athletes [312], with an additional influence of MC on GIs. GIs worsen in most healthy young women either during LaL or EaF [313,314,315], with higher reports of stomach pain at menses than at other MC phases, especially in dysmenorrheic women [314]. During L, women excrete hard stools and have delayed transit, while at the time of menses, stools are looser and more frequent. Studies [314,316] also reported changes in bowel habits in healthy women taking COCs, with more GIs on the first days of menstrual bleeding. However, MC does not appear to alter rectal motility or sensitivity [317,318]. In parallel, intense physical activity induces changes in gut microbiota composition [319,320], which is known to play an important role in athletes’ health, well-being, and sports performance. A possible direct influence of sex hormones on the brain-gut-microbiota axis has been suggested, in view of the effects of PG on gut motility during pregnancy, resulting in constipation, increased reflux, and biliary dysfunction [313].

- Urinary function

Recent reviews [321,322,323,324,325] have highlighted that female athletes show a three-fold higher risk of developing urinary incontinence (UI) than male athletes or female non-athletes. The UI is mainly explained by the imbalance of forces between the abdomen and pelvis [324], with increased intra-abdominal pressure generated during high-impact exercise, which overloads the pelvic organs and creates a pelvic floor dysfunction in high-performance athletes [321,324]. The risk of UI appears dependent on the amount of training and the type of sport, with a prevalence varying from 10% in low-impact to 80% in high-impact sports [323,325,326,327,328,329]. UI may influence sports performance [328] either directly during competition or indirectly by interfering with everyday life or training [323,328]. No studies have investigated the impact of MC or contraceptive use on UI, as it is assumed to be the result of a mechanical effect, but a recent study [329] reported that UI was significantly associated with low energy availability across all sports categories.

In addition, proteinuria and hematuria are common in elite athletes, believed to be benign and transient [330]. The prevalence of proteinuria during exercise (17%) is strictly related to the exercise intensity rather than the duration, whereas hematuria (30%) is influenced by both [330,331]. Most often, they are of renal origin, probably due to a temporary hemodynamic impairment, partially of glomerular but principally of tubular function [332]. Factors contributing to exercise proteinuria and hematuria may include vascular changes, hypoxia, lactate accumulation, oxidant stress, foot-strike, bladder trauma, and hormonal changes [333]. No effect of sex or hormonal status appears to occur [330].

### 3.5. Endocrine Response, Metabolism and Immunity

As mentioned in Section 2.1., very few studies directly exploring the impact of hormonal status on performance and metabolism have been conducted on elite athletes. To identify the potential mechanisms, we here present the research on endocrine response, metabolism, and immunity, although without performance being directly explored. Female athletes with hyperandrogenism linked to either polycystic ovary syndrome or differences/disorders of sex development are not included.

#### 3.5.1. Anabolic Hormones

- NMC women (Table 3)

There is no consensus on whether MC phases influence TES concentrations, either at rest or during exercise [186,334,335,336,337,338]. Regardless of the MC phase, it is important to note that basal TES values were higher in elite female athletes than non-elites, with a more marked exercise response, which could indicate greater capacities for higher work rates and recovery [339,340]. Regarding GH, there is a wide consensus that basal and/or exercise GH response is higher at PeO vs. the other MC phases [38,341,342,343,344], associated with higher E2 levels that facilitate the central drive of pulsatile GH secretion. The data appear less clear for IGF-1 [342,343,344,345]. Gleeson et al. [344] hypothesized that the effect on IGF-1 levels was more subtle, with only a modestly elevated baseline IGF-1 from PeO onward despite a two-fold increase in GH secretion due to a reduction in GH sensitivity.

- AM women (Table 3)

The 24-h hormone profiles in AM vs. NMC athletes are characterized by decreased LH pulsatility, with lower TES and higher SHBG concentrations [346]. Most studies have reported increases baseline levels with distorted patterns of GH pulses [36,347,348]. However, studies have reported a similar GH secretion pattern in oligomenorrheic and NMC women exposed to a psychological stressor [349], and no major differences in GH or IGF-1 responses during exercise bouts could be detected in AM compared to NMC endurance athletes recruited from national teams and competitive clubs [65].

- COC women (Table 3)

There is a clear consensus that baseline blood and saliva TES concentrations decrease with COC use [12,335,350,351,352], possibly due to an increase in SHBG concentration, although the extent of the reduction was variable across women [181]. However, Edwards et al. [353] found the same TES increase over the course of the competition, whereas Crewther et al. [351] reported reduced TES responses to training and competition with COCs. From an anabolic viewpoint, this decrease in TES may be compensated by the increase in GH. Indeed, most studies have shown higher GH both at rest and during exercise in temperate or hot environments with COCs [38,348,354], probably because of elevated levels of total estrogens, while there are too few works to conclude on IGF-1 [36,348].

#### 3.5.2. Other Hormones

- NMC women

Cortisol levels at rest and during exercise appear to be independent of the MC phase, with the same increment in response to prolonged submaximal exercise [335,355,356,357,358] as adrenocorticotropic hormone (ACTH) [355]. However, cortisol responses to awakening remain discrepant, with either no change across MC [359,360] or a blunted response in LaL and EaF [361,362]. The relationship between changes in E2 and PG and thyrotropin (TSH), T3, and PRL concentrations with or without stimulation has been investigated, with little or no effect observed [363,364]. Submaximal exercise elicits increases in plasma arginine, vasopressin, and renin independent of MC [355,365], but aldosterone appears significantly elevated during MiL [365]. MC does not affect beta-endorphin, insulin, C-peptide, amylin, glucagon, leptin, or adiponectin, with the same responses to prolonged exercise during EaF and MiL [58,358,366].

- AM women

Most [347,348,357,367,368,369,370] but not all studies [65,371,372] have shown higher basal cortisol levels in AM vs. NMC athletes, with inverse associations between cortisol and LH [370]. This mild hypercortisolism could be due to extrapituitary modulators of adrenal responsiveness to ACTH [373] and interpreted as a redistribution of adrenal steroid metabolism in favor of glucocorticoid production to ensure adequate blood glucose levels in a condition of energy deficiency [368]. Cortisol responses to exercise, however, appeared blunted in most studies [372,373], possibly due to a normal limitation of adrenal secretory capacity [373]. In contrast, some studies observed either a larger cortisol increment in AM athletes in response to exercise [357] or the same response as in NMC athletes [65,374], despite a smaller cumulative ACTH response in the CRF test, which was interpreted as functional changes due to intensive training [374]. Several authors have reported decreased total T3 in AM athletes, coupled with lower energy intake and availability [31,32,33], increased insulin sensitivity and decreased insulin levels [372,373,375], providing a mechanism through which adipose tissue detects energy deficiency and, in turn, downregulates leptin gene expression and secretion, with parallel hypoleptinemia [375]. There is, however, no consensus for PRL. Indeed, normal basal and stimulated PRL levels were found in women runners with menstrual dysfunction [376]. In contrast, Rickenlund et al. [36] reported that the 24-h hormone profiles in AM athletes were characterized by a decreased peak amplitude of PRL, and in some studies, but not all [65], the failure of PRL to increase in response to exercise has been highlighted [369,372], possibly due to the lack of E2 [372]. Hothari et al. [374] but not Meyer et al. [377] reported higher basal beta-endorphin in AM subjects, whereas the response to exercise was similar in AM and NMC athletes [366,377,378], suggesting that the MC alterations in AM athletes are probably not due to an increase in opioid tone. Last, resting levels of aldosterone seemed to be higher [365], and a reduced adrenergic response to intense exercise was shown in AM athletes [371] that might decrease performance by reducing the sympathetic drive essential for the cardiovascular and metabolic adjustments needed.

- COC women

COC use has been associated with increased resting blood, saliva cortisol, or urinary cortisol [350,356,379], with similar change [350] or a blunted response [351,354,356] to exercise, with or without a training effect. However, other studies observed no change in cortisol levels in moderately trained subjects or elite female athletes [335,380,381], either at rest or after exercise, indicating a similar immune-endocrine function in both temperate and hot environments [381]. Weeke et al. [364] reported an increase in TSH with COC vs. NMC, with similar fasting plasma glucose, C-peptide, insulin, and adiponectin levels [58,382]. Yet, Rickenlund et al. [348] reported that COC treatment was able to correct the lower levels of insulin in endurance athletes with menstrual disturbance, suggesting that COCs improve metabolic balance in AM athletes.

#### 3.5.3. Metabolism and Immunity

- NMC women

The literature suggests that E2 may alter carbohydrate (CHO), fat and protein metabolism antagonistically to PG, which often appears to promote proteolysis and lipolysis. In parallel, both E2 and PG were reported to suppress gluconeogenic output during exercise [383]. Some studies have thus reported greater lipid oxidation in L, with higher glucose rates of appearance and disappearance during exercise in F than in L [384,385]. These effects were found to be blunted or completely annulated by CHO supplementation, however, during prolonged exercise [384,386]. Other works have shown that maximal peripheral lipolysis during exercise was not significantly affected across MC, either at rest or during moderate exercise [387], with no change in substrate oxidation in NMC females in EaF, MiF, and MiL [388,389]. Both worse [390] and better [391] running economies in L vs. F have been reported. In the same way, data on blood markers of muscle damage and inflammation [creatine kinase (CK), myoglobin, lactate dehydrogenase, IL-6, tumor necrosis factor alpha (TNFα), and CRP are discrepant in well-trained women, with [350] or without [392,393] an impact of MC phase, with higher concentrations of pro-inflammatory interleukins in PMS vs. non-PMS women [45]. Only few studies investigated the variations in immunity in trained NMC female runners without respiratory disorders and reported no significant change across MC [293,394].

- AM women

No differences in ratings of perceived exertion or plasma lactate were found between AM and NMC runners following maximal and submaximal exercise tests [256], whereas blood glucose was either decreased [28] or not [371] in AM vs. NMC athletes. Data on inflammatory markers and endothelial function (TNFα, IL-6, CRP, CK, soluble vascular adhesion molecule-1, cholesterol, triglycerides, glutathione peroxidase, and reductase) are discrepant with no change [379], or altered [395,396] or improved [397] values in AM vs. NMC subjects. Last, lower saliva IgA levels were observed in AM vs. NMC athletes [295].

- COC women

Some authors have explored performance and metabolic responses to COCs in parallel, with mixed results [31,33,35,37,38]. Studies not exploring physical performance have also reported divergent results with COCs, with either no decreases in overall CHO and lipid oxidation rates [398] or an increase in fat oxidation during prolonged exercise with a concomitant rise in lipolytic hormones [354,389,399,400], suggesting a preference for lipid metabolism by skeletal muscle in COC vs. NMC women, although this disappeared during heavy exercise. Moreover, it seems that the fed or fasting conditions for exercise performance have a greater impact on substrate oxidation than COCs [400]. In some studies, COC use was reported to increase total and LDL cholesterol [30,36,67,379], with elevated chronic low-grade inflammation and oxidative stress that could be detrimental to physical activity and elevate cardiovascular risk [401], such as basal CRP [379,380] alone [380] or combined with TNFα [379]. During exercise, an increase in CK [402,403] and a decrease in IL-6 with COCs vs. F was reported in moderate-level athletes [350], although this was blunted in elite female athletes [380]. No other cytokine change was found in either temperate or hot conditions [381], but COC users vs. non-users were reported to have an impaired sweating onset threshold and thermosensitivity [404].

### 3.6. Anti-Doping: Athlete Biological Passport (ABP)

The World Anti-Doping Agency (WADA) officially implemented the ABP in 2009 to monitor variations in defined biomarkers over time [405]. The ABP has three modules: hematological, steroidal, and endocrinological. The hematological module was the first to be introduced, with the aim of detecting any form of blood doping. The steroidal module was launched in 2014 with the aim of detecting doping with anabolic agents. The third endocrinological module to detect doping with growth factors has not yet been implemented. The objective of the passport is to provide a profile of the individual athlete’s variables as a guide for detecting doping practices. Its strength is the personalized threshold values calculated for each athlete on the basis of population data and the previous results obtained for this athlete, which allows for precise monitoring of markers. All the results from anti-doping tests are recorded, and the passports are generated on the dedicated ADAMS (Anti-Doping Administrative & Management System) platform. The construction of these modules was based on the study of multiparametric markers obtained in great part from the data from large population studies [406,407].

#### 3.6.1. ABP: Steroidal Module

- NMC women

Since the urine concentrations of steroidal hormones such as testosterone (T), epitestosterone (E), androsterone (A), etiocholanolone (Etio), 5α-androstane-3α,17β-diol (5αAdiol) and 5β-androstane-3α,17β-diol (5βAdiol) are recorded for the establishment of the ABP, understanding the natural hormonal fluctuations in female athletes is of the utmost importance especially given the physiological phenomena that can affect these biomarkers. Indeed, Kuuraane et al. [408] pointed out that sex has an effect, with lower concentrations of these biomarkers in females compared to males. Mareck-Engelke et al. [409,410] performed the first studies in female subjects and established that one of the most sensitive markers, the T/E ratio, was significantly dependent on MC. This observation was more recently confirmed by other authors [411,412], who studied urinary steroid profiles throughout the MC in healthy female volunteers and concluded that E was one of the biomarkers that fluctuated the most. In addition, Schulze et al. [412] established that T and its metabolites exhibited higher concentrations in PeO, with similar variations over two consecutive MCs, but inter-individual variations remained very high. Recently, Mullen et al. [413] demonstrated that the variability of the ABP biomarkers in Swedish and Norwegian athletes was higher in females compared to males. Last, Knutsson et al. [414] observed lower levels of E in PeO and higher levels of 5αAdiol in L in women with moderate to high self-reported levels of recreational physical activity.

- COC women

Studies have been performed with COC and emergency (i.e., progestin-only, levonorgestrel) contraceptives in healthy women [415] or elite athletes [416]. One of the main results was that E concentrations were significantly lower in COC users vs. non-users, whereas T showed no difference, leading to a higher T/E ratio. No impact was observed on the A/Etio ratio or the 5αAdiol/5βAdiol ratio. With emergency contraceptives tested in few subjects, significantly lower concentrations were observed for E, A, Etio, and 5βAdiol, although none of the ratios monitored in the ABP changed significantly.

- Women with other drug administration

The intake of some drugs can impact the steroid profile, the most studied substance in this field being anabolic androgenic steroids (AAS). Buisson et al. [417] investigated the effect of oral intake of 100 mg DHEA per day over 4 weeks for 11 female subjects training regularly. The ADAMS platform indicated that 10 of the 11 passports were clearly suspicious, with most of the ratios exceeding the individual threshold. Knutsson et al. [414] studied in 24 women the impact of 10 weeks of treatment with 10 mg testosterone cream and reported atypical results, mainly for the T/E ratio in 6 subjects, with only 2 subjects having an atypical 5αAdiol/E ratio [418,419]. Sometimes, athletes can only be tested once, and in such cases, the interpretation of a stand-alone data item can be complicated, especially for female athletes. For example, in women self-reporting the use of enanthate testosterone gel [420] or in testosterone doping cases [421], the urine steroid profile could not be considered suspicious in view of the single test results, but the serum samples lead to higher suspicious TES concentrations. In addition, a few works have been performed on other prohibited substances [422], such as nonsteroidal aromatase inhibitors (letrozole, aminoglutethimide, and anastrozole), showing an increased concentration of all steroid profile parameters for at least one month, but not enough to indicate doping.

#### 3.6.2. ABP: Hematological Module

For the purpose of detecting any form of blood doping, 12 blood parameters are recorded on the ADAMS platform for each doping test performed on athletes: hematocrit, hemoglobin (HBG), immature reticulocyte fraction, mean corpuscular hemoglobin, mean corpuscular hemoglobin concentration, mean corpuscular volume, platelets, red blood cell count, red cell distribution width, reticulocyte count, reticulocytes percentage (RET%), and white blood cells (TD2019BAR). HBG and RET% remain the main biomarkers monitored longitudinally, as well as two scores: the OFF-hr Score (OFFS) and the Abnormal Blood Profile Score (ABPS) calculated automatically for each test result [423,424].

- NMC women

Only one study examined the impact of MC in healthy women [425]. Lower RET% in F vs. PeO and L was found, with no difference in HBG, hematocrit, and red blood cell count. The authors concluded that most of the module parameters were stable throughout MC, but they emphasized the importance of information on iron supplementation when evaluating hematological passports. It should be noted, however, that no study to date has been conducted on female athletes and that none has investigated the impact of AM or COC use.

- Women with other drug administration

One study [426] investigated the impact of MC on the GH biomarkers after administration of growth hormone-releasing hormone (GHRH) in young, healthy NMC women vs. men. Whereas IGF-1 provided the most promising results to detect GHRH administration in male subjects, monitoring the women’s GH biomarkers longitudinally appeared more challenging because of the much greater intra-individual variation probably linked to the hormonal status.

## 4. Practical Considerations and Perspectives

### 4.1. Summary of Current Knowledge and Limitations

#### 4.1.1. Impact of Hormonal Status on Physical and Cognitive Performance

Most of the studies on elite female athletes have shown no significant change in physical performance across MC, although it should be noted that no study tested prolonged endurance exercise. Most of them reported a better mood with high E2 levels, with a correlation between PG levels and subjective anxiety and a huge negative effect in LaL and EaF in women suffering from PMS. However, there is no consensus, as a wide range of inter-individual responses was noted, and most works failed to directly investigate in parallel the impact of the MC phase on performance and psychological mood states. There is no study on the MC impact on postural and motor control in elite athletes. Regarding AM athletes, it is not possible to conclude on the potential direct short- and medium-term effects of the lack of E2 and PG, and altered endurance or resistance, mood, coordination, and memory performance may occur. The results on COC use have not been conclusive, whatever the type of exercise, as various central or peripheral changes have not been confirmed or correlated with performances over time. Similarly, no conclusions can be drawn on the emotion-related positive or negative effects of COC. It should be noted that only the effects of COCs have been studied, most often in monophasic administration, yet increasingly more athletes are using bi- or triphasic COCs or progestin-only contraceptives.

#### 4.1.2. Impact of Hormonal Status on Health Risks

LaL and EaF, when E2 and PG concentrations are at their lowest, may be deleterious to some of the mental and/or physical health parameters, whereas high PG levels might limit the risk of ACL injuries. Yet caution is required until all these findings are specifically validated in the elite athlete population. Both food intake and fat mass are known to be greatly decreased in many AM athletes, often with parallel lower fat-free mass. In NMC and COC athletes, data are much more inconclusive, and the mechanisms remain unelucidated. The very low E2 levels in AM athletes predispose them to a greater risk to bone health, which nevertheless appears blunted by high-strain physical training. The early initiation of COC use may alter bone health, but most studies indicate a possible beneficial effect of hormonal contraception in AM athletes, although this depends on the administration route. In addition, COC use seems to reduce the risk of ACL injury because of the progestative component, although no clear conclusions can be drawn at this time. The limited studies on the impact of hormonal status on visuospatial abilities, verbal skills, executive functions, and postural and motor control have been inconclusive for sedentary or recreationally trained women. The data to date, however, do not rule out or contradict the possibility that E2 and PG influence cognitive and motor function in elite athletes, who are confronted with much more difficult and highly specific tasks. Part of cardiovascular function is unquestionably modulated by hormonal status. Indeed, although baroreflex sensitivity does not change with hormonal status in NMC, AM, or COC women, resting HVR appears modified during L with an increased sympathetic tone, probably resulting from an altered balance between E2 and PG. There are, however, contradictory data with COCs and too few studies during exercise. No change in VO_2_max across MC was noted in young, healthy women without respiratory disorders, and the higher ventilatory drive observed in L in untrained subjects is blunted with training. There is consensus regarding the negative repercussions of L and COC use on asthma in sedentary and recreational women, with the involvement of PG and, to a lesser extent, E2, but studies are still needed in elite female athletes, as the effects of AM on EIB and URTI remain unknown. Although female athletes often experience alterations in urinary function, this is unrelated to hormonal status, in contrast to gastrointestinal function, which is significantly impacted in LaL and EaF. Regarding the endocrine and metabolic impact of MC, it clearly does not influence basal cortisol levels, whereas GH levels are increased via E2 at ovulation. There is no consensus for the other hormones because of either the contradictory results or the insufficient number of studies. Similarly, and contrary to an ingrained belief, there is no consensus on significant metabolic changes between F and L and with COC intake, with only small differences, if any, in a fasting state, which seems to be offset by food intake. In AM athletes with energy deficiency, there is a drop in basal T3, insulin, leptin, and IgA but with metabolism more adapted to the lack of sex hormones than actually altered. TES levels are significantly lowered with COCs, with most studies showing an increase in GH concentrations, probably thanks to their estrogen component. CRP and CK levels appear to be increased under COC use, but mechanisms remain to be determined, and there are too many discrepancies for the other hormonal or metabolic markers to conclude. Last, the impact of hormonal status on the steroidal module of the ABP is significant for the T/E ratio, with only one study on the hematological module. Given the possible confounding factors, new investigations in elite athletes are essential to confirm and interpret the ABP data, which was mainly obtained in healthy sedentary women.

#### 4.1.3. Limitations

As we have seen, a consensus has been difficult to reach on most of the results, regardless of the parameters, and it is therefore difficult/impossible to draw strong conclusions. We identified the major methodological shortcomings that might influence the strength of the deductions and limit the current knowledge.

First, it must be highlighted that: (i) many studies were performed with extremely small sample sizes, significantly limiting their power, especially given the very wide inter-individual variations in E2 and PG concentrations; (ii) reproducibility between cycles was rarely studied; (iii) many studies followed cross-sectional designs with sometimes no or an inadequate control group, which can strongly bias the results because variability in hormone levels and physical or cognitive capacity cannot be accounted for.

Second, comparisons were often made: (i) without checking the exact time in the phase for the E2 and PG measurements, despite the great variability of MC duration, particularly with a shortened luteal phase; (ii) without sufficient precision, often with only one data point in F and one in L; (iii) without taking into account whether or not a participant suffered from PMS; (iv) using mean values, which mask individual effects regarding who is more impacted and therefore more at risk; (v) without controlling for the great diversity of estrogen/progestin doses and the progestative molecules of the different COCs, all of which are parameters that can interfere with the results; and (vi) without taking into account progestin-only use, either oral or IUD.

Third, (i) composite scores for cognitive tasks may have prevented the detection of specific effects on cognitive processes; and (ii) tasks that were too easy for healthy young women, who generally have high cognitive capacity, may have resulted in a ceiling effect.

Fourth, the very limited number of studies on the influence of hormonal status on the performance and health of the very specific population of elite athletes is surprising.

### 4.2. New Approach to Ensure Optimal Performance and Health

In the future, studies should include large panels of elite female athletes and ensure objective and precise measurements of the different moments of the follicular and luteal phases (EaF, MiF, LaF, PeO, EaL, MiL, LaL), taking into account the extended or shortened durations of these phases. Other factors that should be included are the presence or not of PMS and the doses and molecules of hormonal contraception, if any.

We here present lines of research that we think should be priorities and that would benefit from a multidisciplinary approach that takes into account the athlete’s environment. Indeed, it is obvious that the impact of hormonal status on the female athlete’s life can only be understood and taken into account when it is considered within the context of a specific sports discipline, with simultaneous analyses of the cognitive, emotional, and physical markers, using multidisciplinary methods that break down the barriers between field and laboratory studies. This research will permit the development of an innovative and personalized approach not only to ensure the optimal performance of elite female athletes on D-Day but also to maintain their good health during and after their careers. It therefore appears crucial to set up a longitudinal battery of complementary physical, psychological and sensorimotor tests, along with questionnaires, to determine as accurately as possible the physical and mental state corresponding to each hormonal status in order to optimize the performances and health of elite female athletes, whether or not they suffer from PMS or airway disorders. In addition, specific elicitation interviews might be proposed to address emotional variations, whether connoted positively or negatively by the athlete herself [427], in order to provide insight into the articulations/transitions between emotions and actions [428]. This situated activity-based approach used not only in simulated but also in real-life sports situations [429,430,431] analyzes the meanings of situations from the athlete’s point of view from a narrative of experience in its actual dynamics and complexity. By examining decision-making and technical or operational choices, it would therefore be possible to determine the effective strategies that elite athletes use. The effect of hormonal status has never been explored, but it seems likely that it modulates benchmarks used in action. This could result in inappropriate technical decisions during the execution of an action, thus decreasing the performance of elite female athletes at certain hormonal phases. Other factors in the environment of elite female athletes have also been neglected and yet are crucial. Indeed, responses to the training load may depend on the MC phase. To optimize the training load for maximal performance, i.e., limiting the risk of overreaching/overtraining or injuries, the first step is to accurately quantify the load. Particular attention should be paid to the environments that are increasingly popular with athletes of both genders, such as training at altitude and hypoxia. Competitions, especially when held in extreme conditions, as in ultra-endurance running [432] and cold-water swimming [433], may potentiate hormonal status repercussions in the female athlete through the additional psychological/physiological stress. Exposure to pollution such as particulate matter 10μm (PM10), nitrogen dioxide (NO_2_), fluorinated organic compounds, and pesticides has also recently been suggested to perturb women’s follicular phase duration, cycle regularity, and fertility [434,435,436]. As endurance athletes are increasingly exposed to these pollutants through inhalation during training or routes such as clothing and food/drink packaging, it is essential to take them into account in future studies on MC disruption and hormonal variations in female athletes over the course of their sports careers. Similarly, although numerous studies have focused on the insufficient energy intake of AM athletes, curiously few have focused on the food intake of female athletes who are not considered as energy deficient. However, nutritional intake is certainly essential and directly linked to the metabolism of these athletes during the cycle or with COC use. It therefore may be necessary to strictly monitor both food intake and metabolism in NMC and COC athletes, without overlooking the microbiota, which probably has a non-negligible impact on mental and physical health, performance and the ABP. Moreover, the evolution of EIB and the prevalence of the use of beta-agonists and glucocorticoids based on the hormonal status of elite female athletes should be investigated, as they may affect their physical and psychological performances and health in the short, medium or long term and may also be confounding factors on their ABP.

## 5. Conclusions

As pointed out in this review, the current knowledge on elite female athletes remains sparse and discrepant. A longitudinal multidisciplinary approach may be the best research choice to obtain early, sensitive, and specific tools that are able to determine whether the phase of the cycle, the absence of the cycle, or the use of various contraceptives has an impact on the physical and cognitive performances of elite female athletes. Monitoring would also determine whether these elite athletes have distinct hormone-dependent profiles and would help define new psycho-physiological performance and health markers that take into account the athlete’s environment: training load and competition stress, psychological and emotional state, food intake, and respiratory tract disorders with the use of therapeutic substances.

## Figures and Tables

**Table 1:** Detailed results about motor achievement.

Variables	NMC Women	COC Women
**Postural control**	**No consensus** No change [16,197,198] More instability in EaF [187,193,194] or reverse [188,191,192,195,196,199], with instability found if task is sufficiently difficult (either the task or a change in sensori-input)	**Too few studies** Stabilized posture or better balance [194,206,207] vs. non-COC users
**Motor control**	**No consensus** No change at all [19,209,210] or change in few tasks only [211,212] or in all tasks [213,214,215,216] No impact of training with mixed results in athletes [18,19,212]	**Too few studies** No change across COC cycle and vs. non-COC users [217,218]
**Strength and resistance training**	**No consensus** No change [30,219] ↘ isometric endurance in F [220] or ↗ force in F and PeO [221,222] ↗ limb proprioception in PeO and L [223] **Consensus?** ↗ muscle strength in F with resistance training [186,224,225,226]	**No consensus** ↘ hand grip muscular endurance [227,228] with inhibiting effect on myofibrillar protein synthesis [229] No difference in maximal force-generating capacity, jumping or hopping [18,19,31,230] No endurance or strength changes with training [224,231,232]

Legend: ↗: increase; ↘: decrease.

**Table 2:** Detailed results about cardiovascular responses.

Variables	NMC Women	AM Women	COC Women
**Cardiac function and hemodynamics**	**Rest: No consensus** No change in function and hemodynamics in healthy [235,236] or athletes [237] in temperate/dry/humid hot environments ↗ HR at PeO [238] ↗ systolic and diastolic blood pressure in LaL vs. EaF [239] ↘ HR and diastolic pressure in PMS women [168]	**Too few studies** No change [260]	**Rest: No consensus** ↗ HR and/or systolic blood pressure [235,261] without change in cardiac output or total peripheral resistance [262,263,264] No change [236,262,265]
	**Exercise: No consensus** No change in cardiac function [237,256,257] ↗ HR in L vs. MiF [258] ↗ post-exercise cardiovagal reactivation in EaF with aerobic training [259]		**Exercise: Too few studies** ↗ blood volume, stroke volume, and cardiac output [269] No change in HR [258]
**Autonomic control**	**Consensus** ↗ LF and LF/HF ratio (HRV) in L in most studies [245,246,247,248,249,250,251,252], no change in [244] No change in baroreflex sensitivity in most studies [240,241,242], ↗ in PeO [243]	**Too few studies** No change [260]	**Too few studies** No change in baroreflex sensitivity/HRV at rest in most studies [236,262,266], ↘ baroreflex sensitivity [267] No change at the onset of dynamic exercise [257]

Legend: ↗: increase; ↘: decrease.

**Table 3:** Detailed results about anabolic hormone responses.

	NMC Women	AM Women	COC Women
TES	**Rest: No consensus** No change [334,335,336] or ↗ in PeO [339] **Exercise: No consensus** ↗ or not in F during exercise [186,337] or ↗ in MiL [338]	**Too few studies** ↘ [346]	**Rest: Consensus** ↘ (30–50%) vs. non-users [12,335,350,351,352] **Exercise: No consensus** No change [353] ↘ responses to training and competition [351]
GH	**Consensus** ↗ in PeO [38,341,342,343,344]	**No consensus** ↗ with distorted pattern of pulses [36,347,348] No change vs. NMC to exercise or psychological stressor [349]	**Consensus** ↗ at rest and during exercise in temperate or hot environment [38,348,354]
IGF-1	**No consensus** No change [342,345] ↗ in PeO parallel to GH [343,344]	**Too few studies** No change vs. NMC in exercise levels of endurance athletes [65]	**Too few studies** No change vs. NMC [36,348]

Legend: ↗: increase; ↘: decrease.

## Abbreviations

5αAdiol	urinary 5α-androstane-3α:17β-diol
5βAdiol	urinary 5β-androstane-3α,17β-diol
A	urinary androsterone
ABP	athlete biological passport
ABPS	abnormal blood profil score
ACL	anterior cruciate ligament
ACTH	adrenocorticotropic hormone
AM	amenorrhea
BMD	bone mineral density
CCK	cholecystokinin
CHO	carbohydrate
CK	creatine kinase
CNS	central nervous system
COC	combined oral contraceptive
CRF	cortisol-releasing factor
CRP	C-reactive protein
DHEA	dehydroepiandrosterone
DXA	dual-energy absorptiometry
E	urinary epitestosterone
E2	estradiol
EaF	early follicular phase
EaL	early luteal phase
Etio	urinary etiocholanolone
F	follicular phase
FAT	female athlete triad
FEV_1_	forced expiratory volume in one second
FSH	follicle-stimulating hormone
GI	gastrointestinal symptom
GLP-1	glucagon-like-peptide-1
GH	growth hormone
GHRH	growth hormone-releasing hormone
GnRH	gonadotrophin-releasing hormone
HBG	hemoglobin
HF	high-frequency peak (HRV)
HR	heart rate
HRV	heart rate variability
ICTP	type 1 carboxyterminal telopeptide
IgA	immunoglobulin A
IGF-1	insulin-like growth factor
IGFBP-1	insulin-like growth factor-binding protein-1
IGFBP-3	insulin-like growth factor-binding protein-3
IL-6	interleukin-6
IOC	International Olympic Committee
IUD	intrauterine device
L	luteal phase
LaF	late follicular phase
LaL	late luteal phase
LF	low-frequency peak (HRV)
LH	luteinizing hormone
MC	menstrual cycle
MiF	mid-follicular phase
MiL	mid-luteal phase
NMC	normal menstrual cycle
NO	nitric oxide
OFFS	OFF-hr Score
PaCO_2_	arterial partial pressure of carbon dioxide
PC_20_	provocative concentration of methacholine that results in a 20% drop in FEV1
PeO	peri-ovulatory time
PETCO_2_	end-tidal carbon dioxide
PG	progesterone
PMA	premenstrual asthma
PMS	premenstrual syndrome
POMS	profile of mood state
PRL	prolactin
RED-S	relative energy deficiency in sport
RET%	reticulocytes percentage
SaO_2_	blood oxygen saturation
SHBG	sex hormone-binding globulin
T3	tri-iodothyronine
T	urinary testosterone
T/E	urinary testosterone/epitestosterone ratio
TES	blood testosterone
TNFα	tumor necrosis factor alpha
TSH	thyrotropin
UI	urinary incontinence
URTI	upper respiratory tract infection
VE	ventilation
VE/VCO_2_	carbon dioxide ventilatory equivalent
VE/VO_2_	oxygen ventilatory equivalent
VO_2_	oxygen consumption
VO_2_max	maximal oxygen consumption
